# Stress and addiction: contribution of the corticotropin releasing factor (CRF) system in neuroplasticity

**DOI:** 10.3389/fnmol.2012.00091

**Published:** 2012-09-06

**Authors:** Carolina L. Haass-Koffler, Selena E. Bartlett

**Affiliations:** ^1^Ernest Gallo Clinic and Research Center at the University of California San FranciscoEmeryville, CA, USA; ^2^Clinical Pharmacology and Experimental Therapeutics, School of Medicine, University of California San FranciscoSan Francisco, CA, USA; ^3^Translational Research Institute and the Institute for Health and Biomedical Innovation, Queensland University of TechnologyBrisbane, QLD, Australia

**Keywords:** neuroplasticity, addiction, corticotropin releasing factor system, ethanol, anxiety, stress-induced

## Abstract

Corticotropin releasing factor (CRF) has been shown to induce various behavioral changes related to adaptation to stress. Dysregulation of the CRF system at any point can lead to a variety of psychiatric disorders, including substance use disorders (SUDs). CRF has been associated with stress-induced drug reinforcement. Extensive literature has identified CRF to play an important role in the molecular mechanisms that lead to an increase in susceptibility that precipitates relapse to SUDs. The CRF system has a heterogeneous role in SUDs. It enhances the acute effects of drugs of abuse and is also responsible for the potentiation of drug-induced neuroplasticity evoked during the withdrawal period. We present in this review the brain regions and circuitries where CRF is expressed and may participate in stress-induced drug abuse. Finally, we attempt to evaluate the role of modulating the CRF system as a possible therapeutic strategy for treating the dysregulation of emotional behaviors that result from the acute positive reinforcement of substances of abuse as well as the negative reinforcement produced by withdrawal.

## Introduction

Drug addiction is a chronic condition characterized by periods of abstinence and relapse. The effects of drugs of abuse on brain function have been extensively evaluated with the intention of developing therapies that can prevent relapse and facilitate the treatment of substance use disorders (SUDs). An extensive literature has shown that addictive drugs affect systems that govern reward pathways (mesolimbic dopaminergic pathway), learning and memory processes (hippocampus), emotion (amygdala), and cognitive functions (prefrontal cortex). The reinforcing effects of drug of abuse have been attributed to actions in the limbic system that in turn influence motivational, emotional and affective behaviors (Rezayof et al., 2002; David et al., 2008; Martin et al., 2008; Nielsen et al., 2011; Xue et al., 2012) and for reviews see (Koob, 1992; Pierce and Kumaresan, 2006; Feltenstein and See, 2008). Specifically, the alteration of reward processing (Wise, 1998, 2005) has been identified as a critical factor that leads to an increase in the chance of relapse (Koob and Le Moal, 1997; Everitt et al., 1999; Koob et al., 2004; Everitt and Robbins, 2005). The development of SUDs is a progression that commences with the first exposure to the drug and ends with physiological and psychological dependence.

Although substances of abuse have different mechanisms of action, repeated exposure has been shown to lead to similar neural adaptations. Addiction to any class of drugs has been described as a learning process. Individuals learn associations between the rewarding effects of the drugs and the environmental cues that predict drug availability. Neuroadaptations in areas associated with learning and memory (hippocampus and amygdala) are affected after a single episode of any drug use by influencing synaptic transmission. Following chronic drug use, the compulsive seeking and uncontrollable use leads to long-lasting alterations in synaptic plasticity, such as changes in synaptic strength.

Human studies (Gawin and Kleber, 1986; Wallace, 1989) and experiments with preclinical models (Thatcher-Britton and Koob, 1986; Piazza et al., 1990; Goeders and Guerin, 1994; Kreibich et al., 2009) have identified stress as a critical factor in the drug addiction process, including triggering relapse. Corticotropin releasing factor (CRF) has been implicated in neuroendocrine and behavioral responses to stress (Britton et al., 1982; Koob and Bloom, 1985). It has been shown to be activated during stress-induced drug reinstatement, where it acts to facilitate relapse and increase anxiety during acute and chronic withdrawal (Shaham et al., 1995; Ambrosio et al., 1997; Koob, 1999) and see (Sarnyai et al., 2001; George et al., 2011) for extensive review.

CRF-induced neuroplastic changes have been studied both in mesolimbic brain circuits that include the ventral tegmental area (VTA) and nucleus accumbens (NAcc) (Ungless et al., 2003; Wang et al., 2007a; Hahn et al., 2009) and also in brain regions associated with emotion, such as the amygdala (Fudge and Emiliano, 2003; Pollandt et al., 2006; Fu et al., 2007; Kash et al., 2008; Francesconi et al., 2009). Despite extensive research supporting the role of CRF in drug addiction, the specific participation of CRF on drug-induced synaptic plasticity that leads to relapse remains undetermined.

This review will attempt to examine recent research on the role of CRF and its interaction with drug-mediated synaptic plasticity. The VTA and the amygdalar nuclei where CRF is highly expressed will be described. We will discuss whether CRF facilitates or inhibits synaptic strength from the basal condition. Finally, we will attempt to integrate the neurobiological changes that result from the interaction of substances of abuse with stress to evaluate alternative drug targets for experimental therapeutics to prevent relapse and facilitate the treatment of SUDs.

### Substance use disorders (SUDs) and stress

SUDs are a chronic and relapsing condition characterized by an intense desire for drug intake during the withdrawal period. This craving process leads to a progression from the initial impulsive consumption to a subsequent compulsive and habit forming consumption that result in loss of control in limiting intake and subsequent inability to change the habit developed over time. One of the main challenges in preclinical addiction research has been to elucidate the pathways that lead to the loss of control of drug use and the predisposition to relapse (Koob and Le Moal, 1997). As described by the *Opponent Process Model*, the repetitive use of addictive substances alters the reward circuits by decreasing the intense pleasure state and by increasing the following unpleasant state. After discontinuation of repeated exposure to addictive drugs, compensatory reactions develop that oppose the primary effects of the drug—the withdrawal symptoms. The reduction of the withdrawal symptoms would therefore represent negative reinforcement. The reduction of the unpleasant state of the withdrawal symptoms becomes the major drive in continued drug use. In a simplified view of the *dopamine theory* (Wise, 1978, 2008; Berridge and Robinson, 1998; Everitt and Robbins, 2005; Diana, 2011), the acute euphoric process obtained by binge-intoxication represents the activation of the dopaminergic system, while the negative component resulting from the withdrawal period is marked by the reduction of dopamine function (Tomkins and Sellers, 2001). The introduction of *functional toxicity* (Weiss and Koob, 2001), which is associated with the unpleasant withdrawal state powered by the recruitment of the stress neurotransmitter, CRF, further expanded the *dopamine theory* as it applies to addiction.

### Corticotropin releasing factor (CRF) system

CRF, also known as corticotropin releasing hormone (CRH), has been shown to induce various behavioral changes related to adaptation to stress. Dysregulation of the CRF system at any point can lead to a variety of psychiatric disorders such as depression, obsessive compulsive disorder, post-traumatic stress disorder and SUDs (Cole et al., 1990; Sarnyai et al., 1992, 2001; Cador et al., 1993; Koob and Kreek, 2007; Koob and Le Moal, 2008a). Footshock-induced stress has been shown to be effective in inducing reinstatement of alcohol (Le et al., 1998, 2000; Gass and Olive, 2007; Richards et al., 2008), nicotine (Buczek et al., 1999), cocaine (Erb et al., 1996), opiate and psycostimulants (Lu et al., 2003) and heroin (Shaham et al., 1997) seeking. Specifically CRF has been associated with drug reinstatement (Shaham et al., 1997; Le et al., 2002; Liu and Weiss, 2002; Funk et al., 2006). CRF has also been shown to produce anxiety-like behaviors during withdrawal from chronic ethanol (Baldwin et al., 1991; Overstreet et al., 2004) and may be responsible for persistent vulnerability and eventual relapse.

The CRF system consists of four ligands: CRF, urocortin (UCN) (Vaughan et al., 1995) 1, 2, and 3, two G-protein-coupled receptors (GPCR), CRF-receptor 1 (CRF-R1) and CRF-receptor 2 (CRF-R2), as well as a secreted CRF binding protein (CRF-BP); see Table 1 and (Bale and Vale, 2004) for CRF system review.

**Table 1:** **Corticotropin Releasing Factor (CRF) system**.

**Name**	**Type**	**Receptor binding**	**CNS expression**	**Peripheral expression**	**Involvement in stress response**
CRF	ligand	CRF-R1 > CRF-R2	synthesized in PVN widely distributed	gut, skin, adrenal gland	HPA axis: induces ACTH release outside HPA axis: controls autonomic and behavioral responses
CRF-R1	receptor	–	CC, CB, MS, HIP, VTA, amygdala, pituitary	β cell pancreas	anxiogenic
CRF-R2	receptor	–	RN, LS, HY, CP	heart, GI, lung, skeletal muscle, vasculature	anxiogenic/anxiolytic
CRF-BP	binding protein	–	CC, HY, amygdala, VTA	Plasma, amniotic fluid, placenta, pituitary gland, liver	Periphery: neutralizes CRF CNS: undetermined
UCN 1	ligand	CRF-R1/CRF-R2	EW	GI, testis, cardiac myocytes, thymus, skin, spleen	Periphery: elevated in heart failure (Wright et al., 2009) CNS: modulate excitatory glutamatergic synaptic transmission (Liu et al., 2004)
UCN 2	ligand	CRF-R2	HY, brainstem, spinal cord	heart, blood cells, adrenal gland	central autonomic and appetitive control (Reyes et al., 2001) gender difference in depressive-like behavior (Chen et al., 2006)
UCN 3	ligand	CRF-R2	HY, amygdala	GI, pancreas	energy homeostasis (Li et al., 2007) anxiolytic-like effects (Valdez et al., 2003)

CeA, central nucleus of the amygdala; CB, cerebellum; CC, cerebral cortex; CP, choroid plexus; EW, cell bodies of the Edinger Westphal nucleus; GI, gastrointestinal tract; HIP, hippocampus; HY, hypothalamus; LS, lateral septum; MS, medial septum; OLF, olfactory area; PVN, paraventricular nucleus of the hypothalamus; RN, raphe nuclei.

It was originally identified as a hypothalamic factor responsible for stimulating adrenocorticotropic hormone (ACTH) secretion from the anterior pituitary (Guillemin and Rosenberg, 1955; Saffran et al., 1955) where it stimulates glucocorticoid synthesis and secretion form the adrenal cortex (Turnbull and Rivier, 1997). Its name was established thirty years before its biochemical identification in the 1980's (Vale et al., 1981) while its gene identifier in the National Center for Biotechnology Information (NCBI) is CRH. It is a 4.7-kilo-Dalton (kDa) peptide and consists of 41-amino acid residues. Neurosecretory neurons of the paraventricular nucleus (PVN) of the hypothalamus synthesize CRF (Meloni et al., 2005). CRF is then released into the afferent portal blood vessels to the anterior pituitary gland where it induces ACTH release in the systemic circulation. The hypothalamic-pituitary-adrenal (HPA) axis is regulated by negative feedback from glucocorticoids that activate glucocorticoid receptors specifically in the PVN and hippocampus. CRF is also expressed outside the HPA axis to control autonomic and behavioral responses to stressors (Palkovits et al., 1983; Swanson et al., 1983) including stress-induced reinstatement of drug seeking.

CRF mediates physiological stress responses by activating CRF-R1 and CRF-R2, which are distributed throughout the periphery and the brain (De Souza, 1995; Bale and Vale, 2004). It is believed that the binding of CRF to CRF-Rs is a two-step mechanism. The N-terminus of the receptor initially binds to the C-terminus of CRF, which initiates a rearrangement of the receptor (Grace et al., 2007). The CRF N-terminus contacts the other sites on the receptor to initiate cellular signaling (Vale et al., 1981; Rivier et al., 1984) and consequently activate the G-protein (Nielsen et al., 2000; Grace et al., 2004; Rijkers et al., 2004; Yamada et al., 2004; Hoare, 2005). The CRF system comprises other peptides with structural homology to CRF. UCN 1 shows 45% sequence identity with CRF and binds with high affinity to both CRF receptor subtypes (Perrin et al., 1995), whereas CRF binds with highest affinity to CRF-R1 (Vaughan et al., 1995; Burnett, 2005). UCN 2, also known as stresscopin related peptide, and UCN 3, also known as stresscopin bind specifically to CRF-R2 (Hsu and Hsueh, 2001; Lewis et al., 2001; Reyes et al., 2001).

CRF-R1 has 415 amino acid residues and it is expressed in the periphery and in the CNS (Chang et al., 1993; Chen et al., 1993; Vita et al., 1993; Potter et al., 1994; Tsai-Morris et al., 1996; Sanchez et al., 1999; Van Pett et al., 2000). Chronic stress mediated by activation of CRF-R1 by CRF has been associated with the development of anxiety disorders (Arborelius et al., 1999); CRF-R1 antagonists have been shown to reduce anxiety-like behaviors (Funk et al., 2007). Transgenic mice with deletion of CRF-R1 (CRF-R1 knock out (KO) mice) have reduced reaction to both stress and anxiety, for comprehensive review see (Bale and Vale, 2004). This anxiolytic effect, however may be attributed to the reduction in circulating glucocorticoids in preclinical models (Tronche et al., 1999). A conditional KO mouse line was generated to differentiate the behavioral from the neuroendocrine CRF-mediated CRF-R1 signaling pathways. The selective inactivation of the limbic structures, but not of the HPA system has shown that CRF-R1 modulates anxiety-like behaviors and it is independent of the HPA (Muller et al., 2003). Furthermore, CRF-R1 is thought to increase susceptibility to alcohol relapse behaviors (Hansson et al., 2006; Heilig and Koob, 2007). A recent study evaluated the role of CRF both within and outside the HPA has shown that CRF via CRF-R1 signaling may have opposite effects on stress-related alcohol consumption (Molander et al., 2012).

CRF-R2 has three variants: α,β, and γ. The α is comprised of 411 amino acid residues and the β is comprised of 413–418 amino acid residues. Both are found in the brain and periphery; however, CRF-R2β is predominantly found in the heart and vasculature (Lovenberg et al., 1995a,b; Kimura et al., 2002; Burnett, 2005). The γ variant is a smaller peptide containing only 397 amino acid residues, and is found only in the human brain (Kostich et al., 1998). The precise role of CRF-R2 in the regulation of the stress response is a subject of intense investigation. Genetic mouse model studies with deletion of CRF-R2 (CRF-R2 KO mice) have demonstrated that CRF activation of CRF-R2 can lead to either an increased or decreased response to stressors (Bale et al., 2000, 2002; Coste et al., 2000; Kishimoto et al., 2000).

The lack of specific antisera that support immunohistochemical experiments and the low resolution of ligand binding approaches have limited the studies to elucidate the CRF-Rs distribution and limit the analysis at the mRNA level. To overcome this impediment, a transgenic mouse that reports expression of CRF-R1 with green fluorescent protein (GFP) has been successfully generated providing a novel tool to investigate the role of CRF-R1 signaling in stress adaptation (Justice et al., 2008).

CRF-BP is a water-soluble, 37 kD protein and consists of 322 amino acid residues (Bale and Vale, 2004). It is a secreted glycoprotein, efficiently stored into secretory granules and released into the extracellular space through exocytosis (Blanco et al., 2011). It contains aspargine N-linked-type oligosaccharides that are critical for CRF-BP binding to CRF (Suda et al., 1989). Previous attempts to identify small molecule inhibitors of CRF-BP have produced limited success due in part to the high affinity (picomolar) of CRF binding to CRF-BP (Behan et al., 1995a) and also because CRF-BP full length (FL) is susceptible to autocatalytic proteolysis (Woods et al., 1999). The spontaneous proteolytic cleavage yields a larger N-terminal fragment of 27 kD, CRF-BP (27 kD), which retains the binding site for CRF and a smaller, 9.6 kD C-terminal fragment, CRF-BP (10 kD) (Woods et al., 1999) with no apparent physiological or pathological role. The unique cleavage site in CRF-BP (FL) has been identified between amino acid residues serine 234 and alanine 235. The generation of two fragments has made it extremely difficult to successfully purify sufficient quantities of CRF-BP (FL) to study the physiological properties of the native protein. CRF-BP is distributed in plasma, amniotic and synovial fluid, the placenta, the pituitary gland, the liver, and in several distinct brain regions, including the cerebral cortex, the hippocampus (Behan et al., 1995a), the amygdala (Herringa et al., 2004) and the VTA (Wang and Morales, 2008). In the periphery, circulating CRF-BP neutralizes the physiological actions of CRF (Kemp et al., 1998). Because of the high affinity with CRF, it is believed that CRF-BP plays a buffer role by reducing the amount of free CRF. In the brain, however, CRF-BP is mostly membrane-bound and expressed in different amounts in neurons and neuroglial cells (Behan et al., 1995b). Within neuronal cells, recent findings demonstrated that discrete subpopulations of VTA dopaminergic and γ-aminobutyric acid (GABAergic) neurons express CRF-BP (Wang and Morales, 2008). The physiological role of CRF-BP in the central nervous system (CNS) is still unclear. Additionally, theories suggest the possibility that CRF-BP may assist the clearance of CRF from the body and may also protect CRF from degradation (Seasholtz et al., 2002). Genetic mouse model studies with deletion of CRF-BP (CRF-BP KO mice) have shown there is an increase in anxiety-like behavior (Karolyi et al., 1999). Electrophysiology studies have shown that CRF signals through CRF-R2 to potentiate N-Methyl-D-aspartate (NMDA)-mediated excitatory postsynaptic currents (EPSCs) in the VTA (Ungless et al., 2003). Furthermore, using CRF (6–33), a peptide that competes with CRF at the CRF-BP binding site, but does not bind to CRF-R2, it was shown that it blocked CRF-induced potentiation of NMDAR-mediated EPSCs (Ungless et al., 2003). Taken together, these results suggest that CRF-BP possesses a diverse role in modulating the CRF-system. As described by *in vitro* and *in vivo* studies, purifying human CRF-BP (FL) in sufficient quantities for investigation has not been successful to date (Woods et al., 1997). There have not been any research tools available to characterize the role of CRF-BP in the CNS by expressing CRF-BP on the cell surface. Therefore, it has not been possible to determine whether CRF-BP participates specifically in the CRF-R2 signaling. A summary of the involvement of the CRF binding in addictive behavior is described in Table 2.

**Table 2:** **Involvement of the CRF binding in addictive behaviors**.

CRF-R1 antagonists	Attenuate stress-induced relapse to drug seeking and behavioral changes associated with withdrawal; small molecules and peptides are available for investigation
CRF-R2 antagonists	Regulation of the stress response and addictive behavior is unclear; small molecules and peptides are available for investigation
CRF-BP antagonists	Modulation of neuronal activity may be a target for both drugs of abuse and stress response; only peptides are available for investigation

## Stress-induced drug addiction: CRF-mediated neurotransmission and plasticity

### Reinforcement: ventral tegmental area (VTA) and nucleus accumbens (NAcc)

Addictive drugs have been shown to increase the concentration of dopamine in the NAcc. Furthermore, the increase of dopamine has been associated with the amplification of the hedonic impact of positive reinforcers (Fibiger, 1978; Berridge et al., 1989) and the development of addictive behaviors (Yokel and Wise, 1975; Bonci and Malenka, 1999; Wise, 2008). The NAcc receives input from the VTA and it is thought that this pathway may be responsible not only for the acute pleasure effect of drug intake, but also for the negative reinforcement and the effects of cues on drug-seeking behaviors (Koob and Nestler, 1997).

#### CRF cellular involvement in the VTA

The VTA receives CRF projections mostly from the limbic forebrain and PVN of the hypothalamus (Rodaros et al., 2007) that form glutamatergic synapses and symmetric GABAergic synapses (Tagliaferro and Morales, 2008). The PVN is the site for CRF synthesis (Meloni et al., 2005) and the majority of asymmetric synapses (glutamatergic) are expressed in CRF- and dopaminergic-containing neurons. VTA dopaminergic neurons express CRF-R1 (Van Pett et al., 2000) and a more recent study has shown that the majority of VTA neurons expressing CRF-BP are dopaminergic (Wang and Morales, 2008). The CRF system modulates dopaminergic neurons by activating CRF-R1 and CRF-R2; however, CRF is not only involved in the neuroexcitability of the dopaminergic system. It may also be responsible for modulating excitatory and inhibitory synaptic inputs since the VTA receive inputs from both CRF-glutamatergic- and CRF-GABAergic-containing neurons (Tagliaferro and Morales, 2008) and for review see Borgland et al. (2010).

CRF increases the firing rate of VTA dopaminergic neurons (Korotkova et al., 2006; Wanat et al., 2008) via CRF-R1, and involves the phospholipase C (PLC)–protein kinase C (PKC) signaling pathway with enhancement of *I*_*h*_ (hyperpolarization-activated inward current) (Wanat et al., 2008). CRF can also induce a transient slowly developing potentiation of NMDA-mediated synaptic transmission via CRF-R2 and activation of the PLC-PKC signaling pathway. CRF-R2-mediated potentiation has been shown to require the presence of CRF-BP (Ungless et al., 2003). The mechanism of action of CRF-R2 and CRF-BP is still under investigation as the research tools needed to study CRF-BP and antisera that specifically target CRF-R2 have not been available.

CRF appears to have both excitatory and inhibitory actions on the dopaminergic neurons in the VTA. Studies using cocaine and methamphetamine have shown that the excitatory effect of CRF on dopaminergic neurons involves fast events, for example action potential firing rate and NMDAR-mediated synaptic transmission, while the inhibitory effects of CRF involve slow forms of synaptic transmission that would result in long-term plasticity (Beckstead et al., 2009). Those observations demonstrated that CRF may have different actions on receptors that mediate the synaptic action on dopamine. This cellular mechanism may refine the role of stress by CRF actions on dopamine-mediated behaviors (Beckstead et al., 2009).

As it has been shown that potentiation of CRF-R2, but not CRF-R1, signaling requires the presence of CRF-BP (Ungless et al., 2003), it has been proposed that CRF-BP and CRF-R2 mediate longer-lasting forms of synaptic plasticity (Bonci and Malenka, 1999). Both behavioral sensitization and long-term potentiation (LTP) share many characteristics such as the involvement of NMDAR activation for the induction of LTP in VTA dopaminergic neurons (Bonci and Malenka, 1999; Ungless et al., 2001). As a consequence, it has been suggested that synaptic plasticity at excitatory synapses on VTA dopaminergic neurons may play a principal role in triggering behavioral change. Since NMDAR activation is required for the induction of LTP in VTA dopaminergic neurons, CRF-Rs activation may modulate longer-lasting forms of plasticity (Bonci and Malenka, 1999; Ungless et al., 2001; Bonci and Borgland, 2009).

#### CRF-mediated neurotransmission and plasticity

Synaptic adaptations observed in remodeling of neuronal circuits in addictive drug studies have been shown to have implications in behavior and memory traits that characterize SUDs. The neuroplasticity underlying drug-induced sensitization has produced a growing body of evidence that suggests it may represent the molecular effect that is critical in modulating addictive behaviors and would contribute to stress-induced compulsive behaviors in addiction.

Axon terminals of CRF neurons synapse onto VTA neuronal dendrites (Tagliaferro and Morales, 2008) and it appears that stress affects the CRF release in this region (Wang et al., 2006). Electrophysiological studies have shown that CRF-BP is required for a slowly developing, transient potentiation of NMDAR-mediated synaptic transmission elicited by CRF via CRF-R2 specifically (Ungless et al., 2003). These results have been corroborated by behavioral studies that determined the effectiveness of stress in triggering glutamate and dopamine release in cocaine seeking of drug-experienced rats (Wang et al., 2007b). Using chronic cocaine preclinical models, the study has shown the positive reinforcement associated with CRF, specifically CRF/CRF-R2/CRF-BP interaction with the dopaminergic system. Those findings support additional research efforts to develop novel approaches that probe CRF-BP on the cell surface.

In conclusion, CRF increases VTA glutamatergic synaptic function, which may facilitate VTA burst firing or induction of synaptic plasticity that may result from repeated exposure to drugs of abuse. This process may produce long-term neuroadaptations that alter stress responses and enhance drug seeking. Electrophysiological studies combined with behavioral studies have proposed that previous experience with drugs of abuse may facilitate the ability of stress to drive drug seeking and, therefore, relapse. These results suggest that CRF may be important for drug-evoked synaptic plasticity in VTA dopaminergic neurons and may represent the molecular substrate that explains the anxiety and stress response during withdrawal from substances of abuse.

### Cellular involvement of CRF in the amygdala

The amygdala is believed to be a pivotal brain region for emotional response and it is critical for providing affective salience to sensory information (Adolphs et al., 1994; LeDoux, 2003; Phelps and LeDoux, 2005). Negative affective responses have been studied in specific nuclei of the amygdala by studying the conditioned fear response (Davis, 1992a,b). The amygdala is widely connected to other limbic regions where it participates in integrating sensory and cognitive information (LeDoux, 1992, 1993). Experimental evidence strongly suggests drugs of abuse act on this system and can modify synaptic events especially during withdrawal. While the VTA has been associated with the reinforcing effects of ethanol (Gatto et al., 1994), the activation of the GABAergic system has been associated with alcohol's anxiolytic effect (Frye and Breese, 1982). In addition to the rewarding circuits of the shell of the NAcc, and brain regions activated by pharmacological stressor, such as yohimbine and footshock were found to be specific in the basolateral and central amygdalar nuclei, and the bed nucleus of the stria terminalis (BNST) (Funk et al., 2006). Preclinical studies demonstrated that exposure and withdrawal from ethanol induces functional and biochemical changes in the amygdala of rats, demonstrating that this circuit is involved in long-term increases in anxiety-like behavior following chronic ethanol exposure (Christian et al., 2012).

The amygdala mediates conditioned and unconditioned responses to aversive stimuli (Davis and Whalen, 2001) and it has been investigated using Pavlovian fear conditioning by pairing a conditioned stimulus with an aversive unconditioned stimulus. The re-exposure of the unconditioned stimulus elicits a conditioned fear response derived by the conditioned-unconditioned association (Pitts et al., 2009). The association signal takes place in the basolateral amygdala (BLA) and is then transmitted to the central nucleus of the amygdala (CeA) (McDonald, 1998; Maren, 1999; Davis and Shi, 2000; Pitkanen et al., 2000; Pare et al., 2004). This transmission process involves both positive and negative associations.

All components of the CRF system, CRF, CRF-Rs and CRF-BP are expressed in the amygdala (Potter et al., 1994). Furthermore, the amygdala is a major extrahypothalamic source of CRF-containing neurons (Palkovits et al., 1983; Van Pett et al., 2000). Both BLA and CeA nuclei play a role in the stress response (Richter et al., 1995; Merali et al., 1998; Koob and Heinrichs, 1999). Extensive studies have shown that the CRF system participates in memory consolidation that involves the BLA-CeA circuit (Roozendaal et al., 2002; Hubbard et al., 2007). It has been observed that CRF release in the amygdala is increased during acute withdrawal (Richter and Weiss, 1999); therefore, it has been hypothesized that CRF may modulate drug-evoked synaptic plasticity (Ungless et al., 2001, 2003) and for a recent review, see (Luscher and Malenka, 2011). The neuronal basis for negative reinforcement is less well-understood; however, more recent behavioral studies have shown that CRF is capable of potentiating excitatory synaptic currents via CRF-R1 in the CeA two weeks following withdrawal from cocaine (Pollandt et al., 2006).

A recent study has shown that CRF-R1 specifically possess a bidirectional role in anxiety (Refojo et al., 2011). While deletion of CRF-R1 in the mid brain dopaminergic neurons increases anxiety-like behaviors and reduces dopamine release in the prefrontal cortex, deletion of CRF-R1 in the forebrain glutamanergic neuronal network reduces anxiety and disrupts transmission in the amygdala and hippocampus (Refojo et al., 2011).

The role of CRF was also evaluated extensively in voluntary ethanol consumption using gene expression and genetic variation in preclinical models see (Bjork et al., 2010) for extensive review. In ethanol-exposed animals, ethanol intake was reduced by administration of CRF-R1 antagonist, and tested using pharmacological interventions that reduce anxiety-like behaviors (Logrip et al., 2011; Zorrilla and Koob, 2012). The reduction of ethanol intake was also observed in transgenic mice with deletion of CRF-R1 (CRF-R1 KO) (Chu et al., 2007). CRF-R1 antagonists reduce drug withdrawal-associated anxiety and attenuate the negative reinforcing effects of ethanol associated with prolonged ethanol exposure (Ghitza et al., 2006; Marinelli et al., 2007; Li et al., 2007; Koob and Le Moal, 2008b; Richards et al., 2008). CRF-R1 inhibitors have shown to attenuate stress-induced relapse to cocaine and heroin in trained animals (Shaham et al., 1998) and to reduce stress-induced reinstatement and stress-induced reactivation of conditioned place preference in many addictive drugs (Koob and Zorrilla, 2010).

#### The extended amygdala

Among the extrahypothalamic structures that contain CRF expressing neurons there is the “extended amygdala.” The extended amygdala is comprised by the BNST, the central medial amygdala (CeA), the sublenticular sustantia innominata and a transition zone forming the posterior part of the NAcc (Heimer and Alheid, 1991). It represents the brain circuit involved in processing the aversive stimuli produced by ethanol withdrawal (Koob and Le Moal, 2001), in which the GABA system has been altered and the CRF system in the adjacent CeA has been shown to be activated (Roberts et al., 1996). Those observations indicate that GABAergic activity within interneurons of the extended amygdala may play a prominent role in the chronic negative emotion-like state of motivational significance for drug seeking in alcohol dependence (Koob and Le Moal, 2001; Koob, 2003, 2009a,b). In addition, an *in situ* hybridization study has shown that recruitment of CRF-R1 signaling, in the components of the extended amygdala, may be responsible of driving the excessive voluntary alcohol intake and may be linked to increase stress activity (Hansson et al., 2007).

The BNST (as well as distinct regions of the CeA) has been associated with stress and anxiety (Walker and Davis, 2008) and is involved specifically with CRF signaling (Davis et al., 1997). The CeA and BNST have direct projections to many brain regions that have been studied to elucidate the symptoms of fear or anxiety (Davis, 1992b). The BNST has been identified as a possible regulator of VTA dopaminergic neuron firing (Georges and Aston-Jones, 2002) and consequently involved in the regulation of acute actions of alcohol, nicotine, and cocaine (Watkins et al., 1999; Carboni et al., 2000; Eiler et al., 2003).

The BNST possesses an extensive network of dopaminergic fibers (Fudge and Emiliano, 2003) and is connected to the reward pathway by extensive projections to the VTA, thus influencing the excitatory input through both NMDA and non-NMDA receptors (Georges and Aston-Jones, 2001, 2002). This dopaminergic excitatory transmission in the VTA requires the presence of CRF (Kash et al., 2008). Acute cocaine administration has been shown to induce dopamine signaling through a specific CRF-R1-dependent enhancement of NMDA excitatory transmission (Kash et al., 2008). This mechanism was described as a short-term form of plasticity in the BNST, which may be responsible for the acute effects of addictive drugs (Kash et al., 2008). These findings suggested that glutamatergic neurotransmission in BNST may be functionally involved with acute reinforcing actions of drug of abuse (Walker and Davis, 2008).

#### Basolateral amygdala (BLA)

The basolateral nucleus of the amygdala (BLA) is critically implicated in emotional learning (LeDoux, 2000), and in reward (Balleine and Killcross, 2006; Tye et al., 2008). Neurons from the BLA project directly to the CeA as well as to the BNST. The BLA is mostly composed by glutamatergic pyramidal neurons and provides the main excitatory input to the CeA and other limbic and cortical structures (Sah et al., 2003); however, the excitatory transmission is believed to be modulated by the relatively small number of GABAergic interneurons found there (Washburn and Moises, 1992). GABAergic interneurons have been identified as regulators of stress and anxiety (Silberman et al., 2009).

CRF is present abundantly in the BLA, in addition to CRF-R1 and CRF-BP, (Sakanaka et al., 1986; Potter et al., 1992; Van Pett et al., 2000); however, the effects of CRF in the BLA have been studied far less than the other nuclei of the amygdala. The BLA has been shown to be a critical nucleus for the consolidation of fear and memory and, therefore, is a possible target for dampening emotional memories. It has been shown that intra BLA infusions of CRF increase anxiety-like behaviors (anorexia and grooming) that are blocked by the administration of a CRF-R1 antagonist (Jochman et al., 2005). Another BLA microinfusion study showed that CRF-R1 activates fear memory consolidation and that this effect is blocked by administration of another CRF-R1 antagonist. The fear memory consolidation process seems specifically regulated by the CRF-R1 activation since CRF-R2 antagonist in the BLA disrupted neither the contextual fear conditioning nor performance of contextual freezing in the drug-free conditioned fear test (Hubbard et al., 2007). BLA CRF-R1 activation has been described as induced synaptic plasticity, and demonstrating that BLA CRF-R1 activation can be pharmacologically blocked by small molecules, the possibility to compromise the consolidation of fear memory suggests a potential therapeutic opportunity to ease the development of intense emotional memories.

#### Central nucleus amygdala (CeA)

The CeA has been identified as locus for both acute positive reinforcement of ethanol self-administration and for the negative reinforcement associated with ethanol withdrawal (Baldwin et al., 1991; Heinrichs et al., 1992, 1995; Koob and Le Moal, 1997, 2001; Zorrilla et al., 2001). The CeA has also been identified as a critical locus for reversing many behavioral effects associated with ethanol intoxication (Hyytia and Koob, 1995).

In the CeA, most neurons are GABAergic (Sun and Cassell, 1993), and CRF is highly co-expressed with GABAergic neurons (Veinante et al., 1997; Day et al., 1999). The CeA abundantly expresses CRF, CRF-R1 and CRF-BP (Sakanaka et al., 1986; Potter et al., 1992; Van Pett et al., 2000). Moreover, in the CeA the action of CRF and ethanol has been shown to increase GABA release (Nie et al., 2004) and the amount of CRF release is increased in preclinical models of ethanol dependence (Merlo Pich et al., 1995). Protein kinase C epsilon (PKCε) has been shown to modulate CRF-R1 signaling in the CeA (Choi et al., 2002) and transgenic mice with deletion of PKCε (PKCε KO mice) have shown reduced anxiety-like behaviors (Hodge et al., 2002). Electrophysiological studies have shown that ethanol-induced GABA release in the amygdala is regulated by CRF-R1 (Nie et al., 2004) and that ethanol-stimulated vesicular GABA release depends on PKCε models (Bajo et al., 2008). PKCε signaling pathway in the CeA is activated by CRF-R1 activation and modulates GABAergic neurotransmission that may contribute to the anxiogenic effects of ethanol (Smith et al., 1998; Timpl et al., 1998). This functional link between ethanol, CRF and PKCε that modulates GABAergic neurotransmission in the CeA may contribute to the dysregulation of emotional behaviors that regulate acute positive reinforcement of ethanol consumption and the negative reinforcement produced by ethanol withdrawal.

It has been shown that there is a critical difference between CRF effects in low/moderate ethanol-exposed animals (binge-like ethanol consumption) and ethanol-dependent animals (chronic-like ethanol exposure). While binge-like ethanol (Lowery-Gionta et al., 2012) may cause transient perturbations of the CRF system which may be able to return to its *homeostatic state*, the chronic-like ethanol exposure (Roberto et al., 2003, 2004) may be responsible for the CRF neuroadaptation that would influence the *allostatic state*. An *allostatic state* is defined as a state of chronic deviation of the regulatory network from their normal process and the establishment of a different set point of “apparent stability” (Koob and Le Moal, 2001). This chronic deviation of reward set point is critically altered during drug withdrawal and may contribute to subsequent neuroadaptation that produces vulnerability to addiction and relapses (Koob and Le Moal, 2001). Acute stress does not increase the mRNA expression of any components of the CRF system in the CeA (Herringa et al., 2004), however, in the CeA of animals exposed to ethanol, there was a significant increase in CRF mRNA expression (Lack et al., 2005) as well as in ethanol-dependent animals during withdrawal (Sommer et al., 2008).

The recruitment of CRF in the CeA during early drinking episodes, before dependence, may initiate neuroplastic changes in the system that may become more intense with additional ethanol exposures (Lowery-Gionta et al., 2012). It has been proposed that this CRF-dependent change contributes to the transition from binge-drinking to ethanol dependence (Lowery-Gionta et al., 2012). The authors also found that ethanol enhances GABAergic transmission in the amygdala at both pre- and post-synaptic sites in ethanol naïve animals, while binge ethanol consumption blunts the CRF-mediated GABAergic transmission (Lowery-Gionta et al., 2012). This study revealed that drinking reduced the effect CRF has on GABAergic transmission. In contrast, others have found that animals dependent on ethanol showed enhanced GABAergic transmission in the CeA (Roberto et al., 2004).

CRF and norepinephrine have been shown to increase GABAergic activity measured by GABA_A_ inhibitory postsynaptic potential (IPSCs) in whole-cell recording from the CeA. This effect was blocked by CRF-R1 antagonists and blocked in CRF-R1 knockout mice (Nie et al., 2004; Kash and Winder, 2006). The augmented GABA release produced by ethanol in the CeA in dependent animals was observed both in electrophysiological and *in vivo* microdialysis experiments (Roberto et al., 2003). Later studies in ethanol-dependent rats corroborated that CRF-alcohol interaction on GABAergic transmission in the CeA is more pronounced during alcohol dependence (Roberto et al., 2004).

## Conclusions

This review has summarized the multiple mechanisms that underlie persistent changes in synaptic efficacy following administration of addictive drugs. It is evident that the CRF system significantly facilitates the induction and maintenance of plasticity in the VTA and amygdala, with resulting enhancement of glutamate-mediated excitation and reduction of GABA-mediated inhibition, thus contributing to the molecular basis of drug addiction.

Neuroplasticity in brain reward circuitry following a history of ethanol dependence has been shown (Hansson et al., 2008). Experimental data illustrated in this review support the hypothesis that stress induces plasticity within the VTA and amygdala nuclei and may participate in the development of a chronic anxiety state that could lead to the development of SUDs. These changes in the limbic neuronal network may represent the trigger that may lead to loss of control of drug use. Addictive drugs have been shown to induce behavioral sensitization and there is a large body of literature that evaluates the role of stress and addictive behaviors. Studies of long-term neuroadaptation in alcohol addiction have shown that brain stress and fear systems become activated (Heilig et al., 2010); however, there is still much to be elucidated pertaining to drugs' actions on the CRF system, both in regard to synaptic plasticity and behavioral responses. Several blood–brain barrier-penetrating CRF-R1 antagonists have been developed, however while some compounds have shown efficacy in animal models to treat alcoholism (Gehlert et al., 2007, 2012), CRF-R1 antagonists have still not succeeded in clinical trials (Koob and Zorrilla, 2012).

Preventing all exposure to substances of abuse is almost impossible, as many psychoactive substances (alcohol, nicotine, caffeine, and prescription medications) are generally accepted in our society. There are many medications that are FDA approved or used off-label for alcohol dependence that focus on the treatment of symptom reduction (disulfuram, naltrexone), assistance with withdrawal (benzodiazepines, valporic acid, varenicline), and relapse prevention (acamprosate, ondansetron, baclofen, topiramate, varenicline, methadone) and others FDA approved medications for other indications are at the preclinical stage (mifepristone) (Simms et al., 2011), however, the recidivism in drug abuse is still a major problem for SUDs. Although different classes of substances of abuse have different mechanisms of action, repeated drug use leads to stimulation of the HPA axis and the abrupt cessation of chronic drug use increases activation of CRF. Medications that modulate stress responses may offer a novel pharmacotherapeutic approach for SUDs. Regulating stress outcomes by acting on the CRF system may offer the possibility to develop that novel therapeutic directed to diminish the effect of CRF in synaptic transmissions. By easing the stress-induced drug seeking, it may be possible to reduce relapse and facilitate the formation of memories with less deleterious behavioral consequences.

### Conflict of interest statement

The authors declare that the research was conducted in the absence of any commercial or financial relationships that could be construed as a potential conflict of interest.
